# Molecular Mechanism Study on the Effect of Microstructural Differences of Octylphenol Polyoxyethylene Ether (OPEO) Surfactants on the Wettability of Anthracite

**DOI:** 10.3390/molecules28124748

**Published:** 2023-06-13

**Authors:** Jiajun Li, Guochao Yan, Shaoqi Kong, Xuyang Bai, Gang Li, Jiawei Zhang

**Affiliations:** College of Mining Engineering, Taiyuan University of Technology, Taiyuan 030024, China; lijiajun1072@link.tyut.edu.cn (J.L.); baixuyang0879@link.tyut.edu.cn (X.B.); ligang1050@link.tyut.edu.cn (G.L.); zhangjiawei1120@link.tyut.edu.cn (J.Z.)

**Keywords:** anthracite coal dust, OPEO-type nonionic surfactant, MD simulation, wettability

## Abstract

Inhalable coal dust poses a serious threat to coal mining safety, air quality, and the health of miners. Therefore, the development of efficient dust suppressants is crucial for addressing this issue. This study evaluated the ability of three high-surface-active OPEO-type nonionic surfactants (OP4, OP9, and OP13) to improve the wetting properties of anthracite via extensive experiments and a molecular simulation and determined the micro-mechanism of different wetting properties. The surface tension results show that OP4 has the lowest surface tension (27.182 mN/m). Contact angle tests and wetting kinetics models suggest that OP4 exhibits the strongest wetting improvement ability on raw coal with the smallest contact angle (20.1°) and the fastest wetting rate. In addition, FTIR and XPS experimental results also reveal that OP4-treated coal surfaces introduce the most hydrophilic elements and groups. UV spectroscopy testing shows that OP4 has the highest adsorption capacity on the coal surface, reaching 133.45 mg/g. The surfactant is adsorbed on the surface and pores of anthracite, while the strong adsorption ability of OP4 results in the least amount of N_2_ adsorption (8.408 cm^3^/g) but the largest specific surface area (1.673 m^2^/g). In addition, the filling behavior and aggregation behavior of surfactants on the anthracite coal surface were observed using SEM. The MD simulation results indicate that OPEO reagents with overly long hydrophilic chains would produce spatial effects on the coal surface. Under the influence of the π-π interaction between the hydrophobic benzene ring and the coal surface, OPEO reagents with fewer ethylene oxide quantities are more prone to adsorb onto the coal surface. Therefore, after the adsorption of OP4, both the polarity and the water molecule adhesion ability of the coal surface are greatly enhanced, which helps to suppress dust production. These results provide important references and a foundation for future designs of efficient compound dust suppressant systems.

## 1. Introduction

Coal is one of the most important energy sources in the world, with global production reaching 8.111 billion tons in 2022. China is the largest coal-producing country in the world, with coal production reaching 4.45 billion tons in 2022 [1,2]. According to data from China’s National Bureau of Statistics, coal energy still dominates China’s consumption structure. By 2030, coal will still be China’s main energy source, with its share expected to exceed 50% [3,4,5]. However, the dust produced by coal production poses a major threat to coal mine safety, air quality, and the health of mine workers [6,7]. In underground coal mining, the operation of coal cutting machines causes dust to accumulate, which, combined with factors such as static electricity sparks and mechanical friction, can easily trigger coal dust explosion accidents [8,9]. Coal-mine-dust-induced respiratory diseases have become some of the most common and serious occupational diseases among coal miners. In a work environment where there is much contact with coal dust, pneumoconiosis can develop into progressive massive fibrosis (PMF), which can be fatal for miners [10,11]. Although countries around the world have implemented strict dust management rules for underground mines [12,13], the potential for an enormous disease burden caused by global coal mining operations means that controlling the hazard posed by dust remains a challenging task. Therefore, dust suppression strategies based on surfactants have gained increasing attention from global researchers as effective methods for controlling coal mine dust levels.

Using water as a wetting agent cannot meet the wetting requirements of highly metamorphic coal, such as anthracite, with an average dust suppression rate lower than 60% [14,15,16]. Fortunately, the continuous development and application of surfactants have provided an effective means for controlling coal dust. Surfactants achieve dust suppression effects via two aspects: on one hand, by reducing the surface tension of the water solvent, surfactants enhance the wetting ability of coal dust [17,18]; on the other hand, surfactants interact with coal dust by means of adsorption [19]. The adsorption of a surfactant solution on the coal surface changes the composition and content of micro-molecular structures in coal, such as carbon–oxygen groups, which is crucial for increasing the wetting ability and accelerating the wetting speed of coal dust.

Traditional methods of surfactant compounding to find the best wetting coal dust solution consume a great amount of resources [5,20]. Therefore, current research focuses on a combination of molecular simulation and experimental characterization to compare the molecular structure characteristics of surfactants and provide further guidance to improve surfactant compounding and application. Among non-ionic surfactants, octylphenol polyoxyethylene ether (OPEO) has a polyoxyethylene chain; is one of the most hydrophilic, dispersion-oriented, and infiltration-oriented agents, with low toxicity and low irritation; and does not harm humans or the environment [21]. To reduce the accumulation of anthracite dust in the air, coal surfaces must have hydrophilicity, and OPEO series agents are ideal choices to obtain this. Many scholars have studied the effects of OPEO surfactants changing the molecular mechanism of wetting low rank coal [22,23,24,25,26,27]; however, there have been very few studies on the micro-adsorption mechanism of OPEO surfactants on anthracite coal surfaces, which indicates a direction for further research.

Most studies have focused on the low-rank coal interface, particularly the lignite interface because lignite already has globally recognized coal molecular models, such as the Wiser or Wender coal molecular models [28]. However, there is a lack of corresponding coal molecular models for anthracite. At present, single-component coal models are the most common tools used to study the molecular dynamics of coal interfaces. Nevertheless, due to coal being a complex multi-component macromolecule, using a single coal molecular model makes it difficult to reflect the true coal situation. Our team has established a high-molecular-weight anthracite model with 55 components via extensive experiments [29]. This model has advantages in the richness of the components and the amount of coal molecules, and it has been applied in many studies [30,31,32,33,34].

In order to investigate the effect of the number of hydrophilic groups of epoxyethane on the wettability of anthracite under the same hydrophobic groups with benzene ring structures, we conducted molecular dynamics simulations and comparative experiments using the octylphenol polyoxyethylene ether surfactants OP4, OP9, and OP13. The comparative experiments included a surface tension test, a contact angle test, an FTIR test, an XPS test, a UV spectrophotometer test, specific surface area and pore size distribution tests, and an SEM test.

## 2. Results and Discussion

### 2.1. Surface Tension Analysis

Surface tension is a fundamental parameter for evaluating the effectiveness of surfactants. The essential condition for wetting coal dust is that the surface tension of the wetting agent must be significantly less than the critical surface tension, which is approximately 45 mN/m [15,35] (independent of coal rank). As shown in Table 1, the surface tension of deionized water is 71.558 mN/m, which is the primary reason for the poor performance of water as a wetting agent.

For non-ionic surfactants in the octylphenol ethoxylate ether (OPEO) group, the critical micelle concentration (CMC) increases with the increase in the epoxy ethane group content [36]. However, when the mass concentration exceeds the CMC, further concentration increases do not significantly affect the number of free monomer molecules [37,38]. At this point, the surfactant molecules will form a monolayer. The mass concentration of the OP series surfactant selected was 300 mg/L, its concentration was higher than the CMC [39,40,41], and the surface tension value remained approximately constant (0.5–1.5 mN/m). Specific results can be found in Table 1.

In the aqueous solutions of the three selected surfactants, OP4 had the lowest surface tension value (27.182 mN/m), with the order of surface tension values being OP4 < OP9 < OP13. The addition of these agents significantly reduced the surface tension of the aqueous solutions. Considering the bending characteristics of the hydrophobic tail chains of surfactant molecules and the effect of the benzene ring structure on their spatial conformation, and with the increase in the number of epoxy ethane groups in the hydrophilic groups, longer hydrophilic chains may bring about spatial effects, limiting the micellization process between surfactant molecules [42,43]. Compared with OP9 and OP13, OP4 has a lower critical micelle concentration (CMC) due to its shorter hydrophilic chain, while the hydrophobic groups are completely identical. According to the diffusion double layer theory, after single surfactant molecules in a high concentration solution form a micelle, the surface of the micelles is further promoted in the rate of micelle formation in the system due to the electrostatic attraction [44,45]. This is also the main reason why OP4 has the lowest surface tension.

### 2.2. Contact Angle Analysis

**Figure 1:**
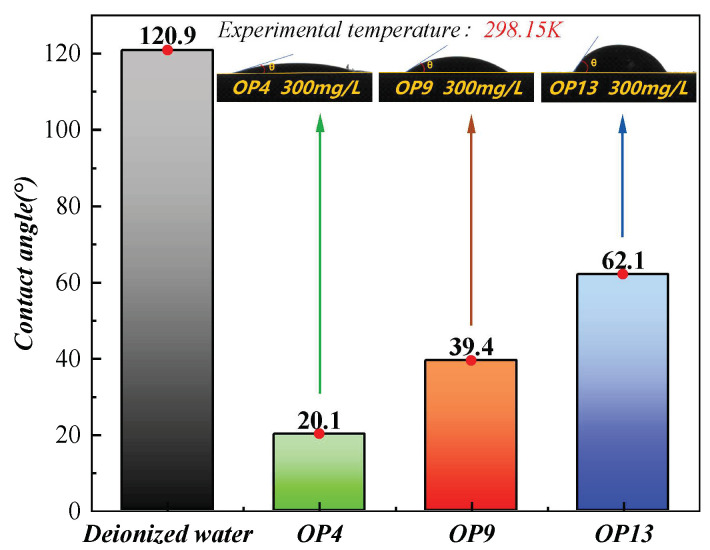
Value of contact angle at 3 s.

The wetting performance of three different surfactant solutions on the surface of anthracite was evaluated by an assessment of the contact angles produced between them [2,46]. Deionized water and coal samples were used as control groups, and their contact angle values at 3 s were compared. On the other hand, as shown in Figure 1, the contact angle between deionized water and anthracite was determined to be 120.9°, clearly highlighting the difficulty of wetting anthracite with pure water. On the other hand, the contact angle values between the coal sample and OP4, OP9, and OP13 were 20.1°, 39.4°, and 62.1°, respectively, with OP4 manifesting the best wetting effect. The results indicate that the introduction of surfactant solutions significantly reduces the contact angle values at the solid–liquid interface while significantly enhancing the wetting ability of the solutions. Therefore, the results are consistent with previous findings on surface tension.

**Figure 2:**
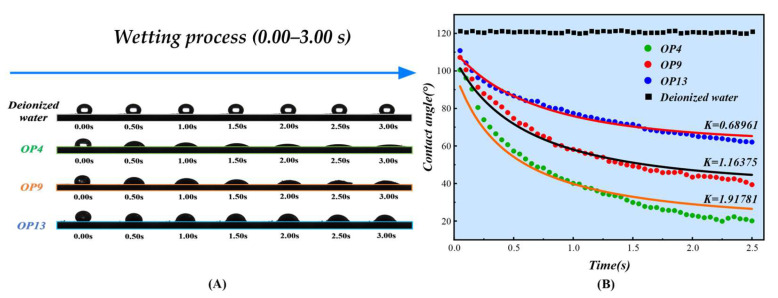
(**A**) Coal dust wetting process. (**B**) Wetting dynamics model.

We studied the wetting process of three surfactants and deionized water on the coal surface within a 0–3 s time interval. Figure 2A illustrates the dynamic nature of the wetting process of coal dust as it varies over time. Combined with contact angle measurement data, the surfactant wetting ability of coal dust is in the following order: OP4 > OP9 > OP13. The experimental group showed a clear contrast with the reference group of deionized water, indicating that the addition of surfactants greatly assists in improving the wettability of anthracite. The wetting process of coal dust can be effectively reflected by using the wetting kinetics model, which can reflect the wetting efficiency of surfactants [47]. For the ideal solid–liquid system, the rate of diffusion and permeation change over time can be expressed by the Equations (1)–(3) below. In the following formula, the *K* value is usually defined as the diffusion–permeation constant, which reflects the wetting efficiency of surfactants. The larger the *K* value, the stronger the surfactant’s ability to wet coal dust:

(1) $$\frac{d\theta }{dt}=-K\theta$$

When permeation and diffusion converge to zero, adding a restriction to the above equation gives Equation (2):

(2) $$\frac{d\theta }{dt}=-K\theta \left(1-\frac{\theta_{i}-\theta }{\theta_{i}-\theta_{e}}\right)$$

Collating the above equation and integrating it yields Equation (3):

(3) $$K=\frac{\left(\theta_{e}-\theta_{i}\right)}{t\theta_{e}}\ln\left(\frac{\theta_{i}\left(1-\theta_{t}\right)-\theta_{e}}{\theta \left(\theta_{e}-\theta_{t}\right)}\right)$$

where $\theta_{t}$ refers to the contact angle value generated at any time within 0–3 s; $\theta_{e}$ refers to the contact angle value at 3 s (when wetting reaches an equilibrium state); $\theta_{i}$ refers to the initial contact angle value generated when the droplet first falls on the coal surface (calculated as 0 s).

We have fitted the wetting process using Equation (3), and the fitted curve is shown in Figure 2B. The fitted equation and relevant parameters are presented in Table 2. According to the data from the graph and the table, the diffusion–permeation parameter *K* value of deionized water wetting coal dust is only 0.01072. The *K* value rapidly increases after adding the surfactant. The *K* values of OP4, OP9, and OP13 are 1.91781, 1.16375, and 0.68961, respectively. Based on the definition of the *K* value, the ability of surfactants to wet coal dust can be ranked as follows: OP4 > OP9 > OP13. This result fully demonstrates the accuracy and reliability of the wetting kinetics model.

### 2.3. FTIR Analysis

**Figure 3:**
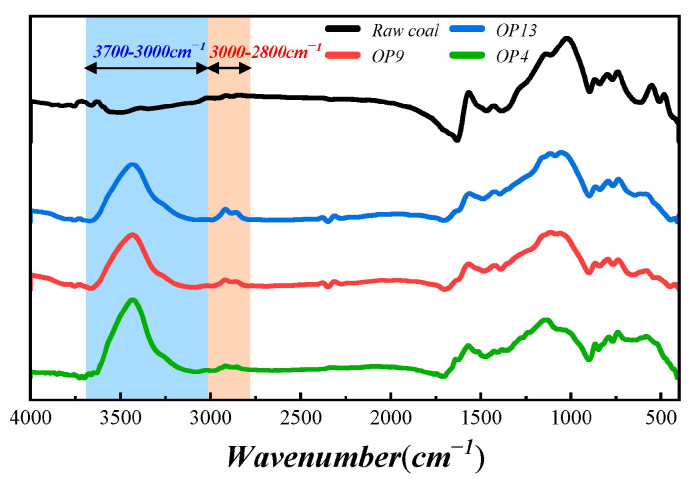
FTIR spectra of different samples.

The surface chemical structure and functional group content distribution of raw coal and three modified coal samples can be characterized and analyzed by FTIR [48]. This approach can explain the micro-mechanism of the increased wettability of modified coal, as shown in Figure 3. By comparing the FTIR spectra of raw coal and modified coal, significant differences were found between the two absorbance bands: the hydroxyl structure absorption band and the characteristic peaks of long-chain alkyl CH_2_ and CH_3_ stretching vibration, which are, respectively, located in the 3700–3000 cm^−1^ and 3000–2800 cm^−1^ regions.

Studies have identified that hydroxyl functional groups, crucial oxygenated functional groups present in coal, primarily serve as adsorption sites for water molecules. Additionally, the majority of hydroxyl groups in coal exist as hydroxyl polymers [49,50]. Nearly 51.53% of the hydroxyl structure adsorption bands in raw coal are filled with the presence of free hydroxyl groups that exist near 3600 cm^−1^ [51]. However, the hydroxyl groups in modified coal have experienced a significant transformation due to the surface modification with OPEO surfactants. This results in the emergence of a robust peak near 3440 cm^−1^, which is not present in raw coal. The fitting results of the characteristic peak in Figure S1 (Supplementary Materials) show that the content of free hydroxyl groups near 3600 cm^−1^ in OP4 is almost negligible, and 5.61% of free hydroxyl groups were observed near 3583 cm^−1^ in OP9, while the content of free hydroxyl groups near 3580 cm^−1^ in OP13 was 6.81%. Furthermore, the modified coal indicates a considerable increase in the OH⋯OH groups content near 3450 cm^−1^ and OH⋯O groups content near 3300 cm^−1^. More specifically, the proportion of OH⋯OH groups in OP4, OP9, and OP13 was 59.57%, 49.32%, and 35.08%, respectively. This indicates that, as the hydrophilic chain length increases, the adsorption amount of surfactant on the surface of anthracite decreases. OP4 can better improve the hydrophilicity of anthracite, and water molecules can develop an extensive binding with the altered coal surface’s oxygen sites by producing a significant number of hydrogen bonds.

The 3000–2800 cm^−1^ region is part of the absorption region for the fat structure. As shown in Figure S2, the most common functional groups in this region are the asymmetric stretching vibration of -CH_2_ near 2920 cm^−1^ and the symmetric stretching vibration of -CH_2_ near 2850 cm^−1^ [52]. Compared with the OP9 and OP13 samples, the OP4 sample has a narrower and less prominent band in the 3000–2800 cm^−1^ range. This is mainly due to the structure of the surfactant itself. As the length of the hydrophilic chain increases, the number of -CH_2_ groups in the surfactant multiplies. In an OP13 molecule, there are 18 additional -CH_2_ groups compared to an OP4 molecule. Therefore, even though the adsorption rate of OP4 is higher than the other two samples, the order of -CH_2_ content is still in the sequence of OP4 < OP9 < OP13 due to the increasing number of -CH_2_ groups in the surfactant.

### 2.4. XPS Analysis

**Figure 4:**
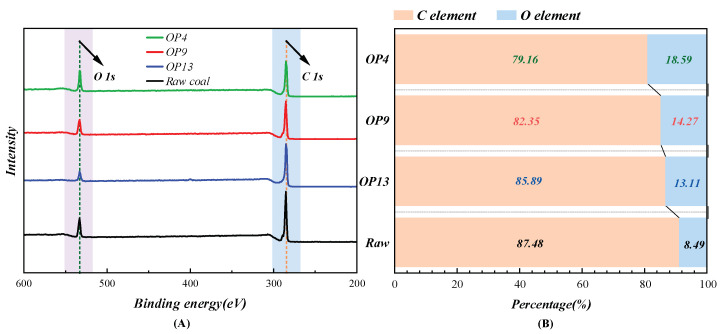
(**A**) XPS spectra of different coal samples; (**B**) proportions of elemental C and O contents in different coal samples.

**Figure 5:**
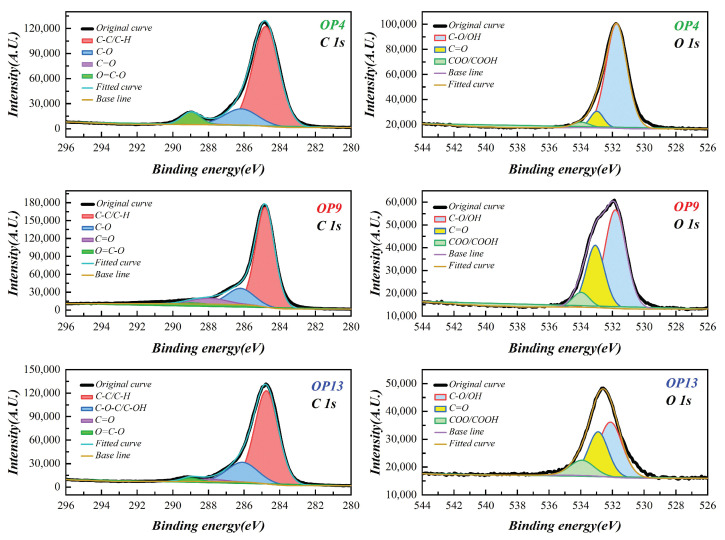
C 1s and O 1s peaks on the surface of three modified coals.

The changes in oxygen-containing functional groups in raw coal and modified coal were investigated using XPS, and it was found that the amount of functional groups on the coal surface significantly affects the wettability [53]. Figure 4A presents the XPS spectra of raw coal and three types of surfactant-treated coal samples. By quantitatively analyzing the C and O contents on the surfaces of raw and modified coal samples based on typical XPS wide-scan total spectra, the results showed in Figure 4B indicate that the carbon content decreased by 8.32%, 5.13%, and 1.59%, while the oxygen content increased by 10.1%, 5.78%, and 4.62% upon the adsorption of OP4, OP9, and OP13, respectively. The surface carbon and oxygen content of modified coal differed significantly after being treated with different surfactants. By fitting the C 1s and O 1s XPS spectra on the surface of the modified coal based on atomic-level differences in electron binding energy, the results were presented in Figure 5 and Table 3.

According to the analysis of C 1s in Table 3, it is found that, after the adsorption of OP4, the content of C-C/C-H groups on the coal surface is the lowest, accounting for only 68.82%, while the content of the C-O oxygen-functional groups is relatively high, accounting for 23.48%. As C-C/C-H groups have strong hydrophobicity and C-O groups are important functional groups for improving coal wettability [54,55], this result suggests that OP4 has a significant enhancing effect on the wettability of anthracite.

Both raw and modified coal surfaces contain three different oxygen atoms with different binding energies. As can be seen from the O 1s spectrum in Table 3, the state of oxygen on the coal surface changed significantly after modification with the surfactant. In the raw coal, oxygen atoms mainly exist in the form of the C=O group, accounting for 59.05% of the total. However, after modification with OPEO-type surfactants, the oxygen atoms on the coal surface transform into the C-O/OH group, which is a strongly polar functional group with high hydrophilicity [56]. Specifically, the content of the C-O/OH group in OP4 even reaches 86.31%, and it exceeds 50% in both OP9 and OP13, becoming the main group of oxygen atoms. In OPEO-type surfactants, the stable π-bond interaction between the benzene ring structure and the aromatic ring structure on the anthracite surface is formed. In this case, the hydrophobic groups are completely identical, and the length of the hydrophilic groups becomes the only factor affecting the surfactant’s large-scale adsorption on the coal surface. Experimental data show that for OPEO surfactants an increase in the length of hydrophilic chains is unfavorable for their full adsorption on the coal surface. Instead, due to the effect of spatial effects, the surfactant molecules cannot fully stretch out, become entangled with each other, and cannot effectively capture water molecules, thus affecting the overall wettability of the modified coal. The results of O 1s indicate that the OP4-modified coal surface has more hydrophilic oxygen functional groups, which gives it better wetting performance.

### 2.5. UV Spectrophotometer Measurement Analysis

**Figure 6:**
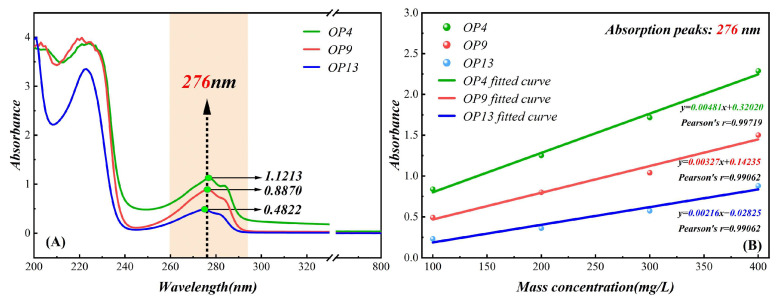
(**A**) UV spectra of three surfactants; (**B**) fitting scales for absorbance and mass concentration of the three surfactants.

UV spectrophotometry is a qualitative or quantitative means of detecting octylphenol polyoxyethylene ether surfactants [57,58], and the adsorption of surfactants on the coal surface can be characterized using differential subtraction. As shown in Figure 6A, the UV absorption spectra of the three surfactants all exhibit strong and weak absorption peaks around 224 nm and 276 nm, respectively, formed by the π-π transition absorption bands of the surfactant benzene ring with auxiliary chromophoric substituents O- and the π-π transition characteristic absorption band of the aromatic compound [59,60]. It can be seen that the absorbance of OP4 is highest around 276 nm, reaching 1.1213, followed by OP9 and OP13 with absorbances of 0.8870 and 0.4822, respectively. The absorbance near 276 nm is generally selected as the fitting baseline for surfactants because the weak absorption peak in this region has better correlation and comparability. The mass concentration–absorbance fitting baseline shown in Figure 6B indicates that the mass concentration of the surfactant solution has a good correlation with absorbance within a concentration range of 0–400 mg/L, and the Pearson correlation coefficient reaches above 0.990, conforming to the Lambert–Beer law [61]. The slope of the fitting line shows that OP4 > OP9 > OP13, indicating that the absorbance of the OP4 solution is more sensitive to changes in mass concentration and has relatively small measurement errors. By measuring the absorbance values of the three types of surfactant solutions adsorbed on coal samples in the upper centrifugal liquid layer and combining them with fitted baseline data, the corresponding mass concentration can be obtained. Then, the adsorption amount of the surfactant on the coal surface can be calculated using the differential subtraction method, represented by Equation (4):

(4) $$w=\frac{\left(C_{0}-C_{1}\right)\times V_{liquid}}{m}$$

where w is the amount of surfactant adsorbed on the surface of the coal sample, mg/g; $C_{0}$ is the initial mass concentration of the surfactant solution, mg/L; $C_{1}$ is the mass concentration of the surfactant solution after adsorption, mg/L; $V_{liquid}$ is the volume of the solution, L; m is the mass of the coal sample, g.

According to Table 4, after averaging the data via repeated experiments, the adsorption capacities of OP4, OP9, and OP13 on the surface of coal were calculated using the difference method and were 133.45, 72.28, and 63.68 mg/g, respectively, showing that OP4 had the highest adsorption capacity. The quantity of hydrophilic and hydrophobic groups and the characteristics of anthracite itself can affect the adsorption ability of surfactants on the coal surface. For OPEO series surfactants, they have the same hydrophobic groups but different lengths of hydrophilic groups, resulting in different adsorption strengths on the surface of anthracite. The experimental results showed that OP4 had the highest adsorption capacity. This was due to the interaction between the hydrophobic groups of OP4 and the hydrophobic sites on the surface of anthracite, which transformed these sites into hydrophilic ones. The hydrophilic head groups of the surfactant extended toward the water, forming hydrogen bonds with water molecules and making it easier to adsorb more water molecules and attach them to the modified coal surface, thereby enhancing the wetting ability of coal dust.

### 2.6. N_2_ Adsorption Curves, Specific Surface Area and Pore Distribution Analysis

**Figure 7:**
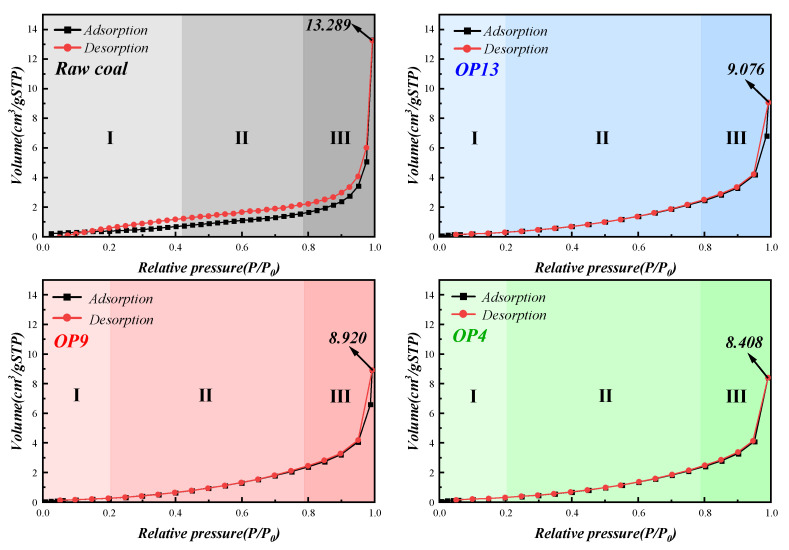
Adsorption/desorption isotherms of coal samples before and after surfactant treatment.

By measuring the N_2_ adsorption/desorption isotherms of coal before and after the adsorption of surfactants, this experiment aims to explore the adsorption mechanism of surfactant molecules on the pore structure of coal samples [62,63,64]. As shown in Figure 7 and classified by Brunauer–Deming–Teller, the adsorption/desorption isotherms exhibit a type II isotherm, indicating the multilayer adsorption of N_2_ [65,66,67]. The adsorption/desorption isotherms can be divided into three stages (with differences between raw and modified coal): Stage I corresponds to P/P0 < 0.20 (P/P0 < 0.42 for raw coal), Stage II corresponds to 0.20 < P/P0 < 0.79 (0.42< P/P0 < 0.79 for raw coal), and Stage III corresponds to 0.79 < P/P0 < 1.

In Stage I, N_2_ adsorbs on coal dust micropores in the form of a monolayer with a relatively low pressure. Compared with raw coal, coal dust treated with surfactants can form a monolayer adsorption at a lower P/P0 and can enter Stage II (the steady rise period) more quickly, and Point B is not prominent compared to raw coal. This indicates that the transition between monolayer and multilayer adsorption in modified coal is smoother, and the coverage of monolayer adsorption and the starting amount of multilayer adsorption will overlap.

Both raw coal and modified coal undergo three stages of adsorption isotherms. The first stage is a starting phase (Stage I), followed by a steady rise phase (Stage II), and finally a sharp increase phase (Stage III). With the increase in relative pressure, a multi-layer adsorption structure gradually forms. When N_2_ approaches the saturation vapor pressure, unlimited adsorption cannot occur. When P/P0 equals 1, the amount of N_2_ adsorbed in the pores of the raw coal is 13.289 cm^3^/g. The amount of N_2_ adsorbed in the pores of the coal samples treated with the three surfactants is 9.076 cm^3^/g (OP13), 8.920 cm^3^/g (OP9), and 8.408 cm^3^/g (OP4), respectively. This indicates that OPEO surfactant molecules not only adsorb on the surface of anthracite via hydrophobic interaction but also penetrate into the interior of the coal pores and are tightly adsorbed in the pore structure of the coal dust surface, resulting in a micropore filling effect. Because of the long hydrophilic chain of OPEO surfactant, surfactant molecules with longer hydrophilic chains could not form a large number of micelles to fill the pores. Therefore, the pores of the three modified coals produce differentiation in N_2_ adsorption.

Another obvious difference is found in Figure 7: there is an H3 type hysteresis loop in the adsorption/desorption curve of the raw coal, reflecting a large number of fissure structures, cracks, and wedge structures in the porous laminar structure of the raw coal [68]. However, no adsorption saturation was observed in the higher relative pressure region. For the modified coal, since the surfactant micelles fill the large and medium-sized pores on the surface of the coal, the adsorption/desorption curves of the three modified coals overlap, and there is no lag phenomenon. This further proves that surfactant molecules can easily enter and greatly adsorb in the pore structure of anthracite, resulting in a large number of mesh structures inside the pore being covered by the surfactant molecules.

The results of the BET analysis showed that the specific surface area of coal dust increased after being treated with surfactant. The specific surface area of the raw coal was 1.365 m^2^/g, while the values of three modified coals, namely, OP13, OP9, and OP4, were 1.548 m^2^/g, 1.602 m^2^/g, and 1.673 m^2^/g, respectively. This phenomenon is attributed to the penetration of surfactant molecules into the pores of coal dust and the enhancement of adsorption and aggregation. Surfactant molecules also indirectly increase the number of micropores on the surface of modified coal by filling the large pores, which leads to an increase in specific surface area. This elevation of specific surface area confirms the increased effective adsorption of surfactant in the pores of the anthracite surface, which leads to the improvement of the wettability of modified coal.

**Figure 8:**
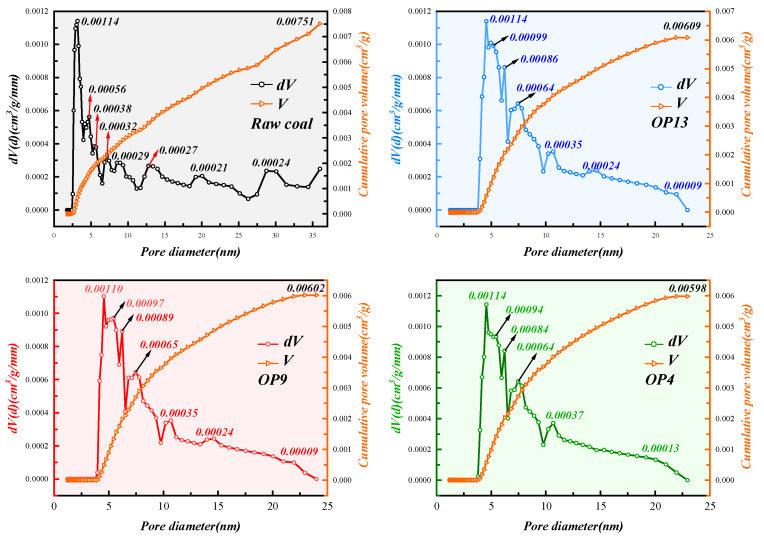
Pore size distribution of coal dust before and after surfactant treatment.

The pore size distribution of coal dust samples was analyzed using DFT [69]. Figure 8 presents the specific pore distribution and cumulative pore volume of raw and modified coal. There were no pores larger than 50 nm in diameter in any coal sample. After treatment with surfactants, the cumulative pore volume was reduced to varying degrees. Raw coal had a cumulative pore volume of 0.00751 cm^3^/g/mm, and the corresponding values for OP4, OP9, and OP13 modified coal were 0.00598, 0.00602, and 0.00609 cm^3^/g/mm, respectively. This indicates that the adsorption of surfactants in the pores reduced the pore volume, with OP4 exhibiting the most significant effect. For the four sample groups, sharp and narrow peaks were observed around 3–4 nm, indicating that most pores had a similar size with a relatively uniform pore type. Only raw coal had some relatively flat and wide peaks in pore sizes of 15 nm or larger, while almost none were present in modified coal. This illustrates that surfactant molecules were closely adsorbed in numerous pores larger than 15 nm in diameter via van der Waals forces, followed by an increase in the number of pores with diameters of 5–10 nm.

### 2.7. SEM Analysis

**Figure 9:**
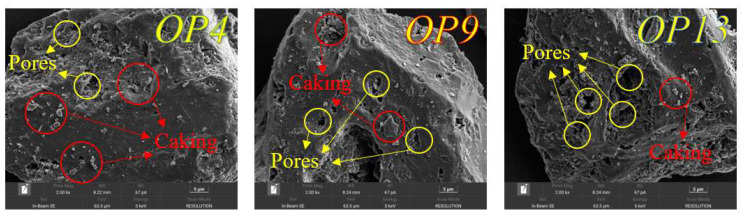
SEM images of coal dust after treatment with three surfactants.

We studied the morphology of three modified coals using scanning electron microscopy. A magnification of 2000× was chosen to ensure the observation of the pore structure on the coal surface. As shown in Figure 9, the pore structure of OP4-modified coal is significantly smaller than that of OP9- and OP13-modified coals due to the “filling effect” of the agent molecules adsorbed on the coal surface. The coal powder agglomerated on the surface of coal samples is called “caking”. These agglomerates, formed by the binding effect of surfactants, will fill larger multi-layer pore structures in raw coal, making the coal dust adhered to the coal surface difficult to remove. In raw coal, the volatile gas film formed between many pores and cracks prevents the water molecules from wetting the coal surface, which inhibits the diffusion of the solution on the coal surface [47]. However, when the pores on the surface of modified coal were filled, more small pores were formed, which promoted the capillary action on the surface of coal dust, leading to the complete infiltration of water molecules and the improvement of the hydrophilicity of the coal surface [70]. Our experiments provide ample evidence for the hypothesis about specific surface area and pore structure distribution.

### 2.8. Molecular Dynamics Simulation Results 

#### 2.8.1. Contact Surface Area

The contact surface area (*CSA*) reflects the strength of adsorption between two materials, and there is a positive correlation between contact surface area and adsorption strength [71]. *CSA* can be calculated using Equation (5):

(5) $$CSA=\left(SASA_{anthracite}+SASA_{surfactant}-SASA_{total}\right)/2$$

where $SASA_{anthracite}$, $SASA_{surfactant}$, and $SASA_{total}$ are the solvent accessible surface area (*SASA*) of the anthracite model, the surfactant, and the anthracite–surfactant binary model, respectively. In this study, the applicable probe radius was 0.14 nm [72].

The *CSA* values of OP4, OP9, and OP13 are 3722.40 Å, 3670.38 Å, and 3442.64 Å, respectively. The highest *CSA* value is observed for OP4, indicating the highest adsorption strength of it to anthracite, while the lowest value was found for OP13. Consequently, it can be concluded that OP4 forms relatively dense network structures on the surface of coal, but longer hydrophilic chains cannot arrange tightly and efficiently due to spatial effects.

#### 2.8.2. Interaction Energy

In the modified coal system, the interaction energy between surfactants and anthracite can be used to measure the strength of adsorption. The more heat released, the greater the negative value of interaction energy, indicating that the structure after adsorption is more stable and the adsorption strength is higher [73]. The total interaction energy *E* is affected by both van der Waals interaction energy *EV* and electrostatic interaction energy *EE*, reflecting the improvement space of modified coal’s wetting performance [74]. The interaction energy is defined by Equations (6)–(8):

(6) $$EV=EV_{total}-EV_{A}-EV_{B}$$

(7) $$EE=EE_{total}-EE_{A}-EE_{B}$$

(8) E=EV+EE

where EV stands for van der Waals adsorption energy; EE stands for electrostatic adsorption energy; E stands for total adsorption energy; $EV_{total}$ and $EE_{total}$ represent the total energy after the adsorption of the two materials is completed; $EV_{A}$ and $EE_{A}$ represent the energy of material A (surfactants); and $EV_{B}$ and $EE_{B}$ represent the energy of material B (anthracite).

It can be seen from Table 5 that through the comparison of the total interaction energy *E*, we found that the OP4-modified coal system had the highest interaction energy and released the most energy during the adsorption process. This indicates that the adsorption configuration of OP4 on the surface of anthracite was more stable, which promoted the enhancement of the surface hydrophilicity of the modified coal. At the same time, it was found that van der Waals interactions always played a dominant role in the three modified coals, which was because OPEO series surfactants belong to the nonionic surfactant family and their adsorption behavior on an anthracite surface belonged to physical adsorption. The OP4 system had the largest negative interaction energy, while the OP13 system with a longer hydrophilic chain had a smaller interaction energy. This indicated that the number of polyethylene units played a decisive role in explaining the adsorption behavior of surfactants. The accumulation degree of OPEO surfactants on the surface of anthracite depended largely on the number of polyethylene units: the more polyethylene units, the larger the volume occupied by the surfactant molecules and the lower the adsorption density.

#### 2.8.3. Relative Concentration Distribution

**Figure 10:**
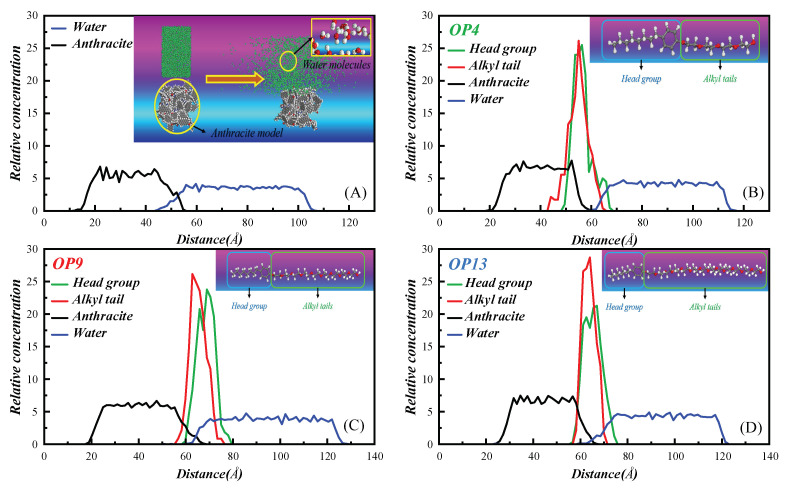
Mass density distributions along the *z*-axis: (**A**) raw coal; (**B**) OP4; (**C**) OP9; (**D**) OP13.

Figure 10 demonstrates the mass density distribution of each component along the *Z*-axis in raw and modified coals, which quantitatively reflects the adsorption ability and adsorption layer structural parameter information of three types of surfactants [37]. Firstly, the initial water layer distance in the raw coal system is 43.38 Å, and the overlap width of coal and water is 12.57 Å. In contrast, in the modified coal system, the initial water layer distances are 59.08 Å (OP4), 59.85 Å (OP9), and 58.19 Å (OP13), with coal and water overlap widths of 1.35 Å (OP4), 5.57 Å (OP9), and 8.84 Å (OP13), respectively. Obviously, in the OP4 system, the overlap between coal and water is minimal, and the contact between the water layer and the surfactant is greater. This implies that OP4 can cover the surface of anthracite more densely, thereby forming a stable intermediate medium at the coal–water interface.

Secondly, the distribution range of the hydrophobic groups of the OP4 surfactant is 44.14–64.51 Å, with a distribution width of 20.37 Å. Within the range of 44.14–60.43 Å, the hydrophobic tail of the surfactant overlaps with the coal surface, with an overlap width of 16.29 Å. In comparison, in OP9 and OP13, their hydrophobic tails, respectively, overlap the coal surface with an overlap width of 10.60 and 8.84 Å. This indicates a stronger interaction between OP4 and the coal surface. The coal surface is mainly composed of hydrophobic sites consisting of numerous alkyl chains and aromatic rings, and there are relatively few hydrophilic sites with negative charges brought by oxygen functional groups. A small amount of surfactant molecules form hydrophilic groups via hydrogen bonding with the few hydrophilic hydroxyl groups on the coal surface. While a large amount of surfactant molecules adsorb to the coal surface primarily via hydrophobic interactions, thus exposing a large number of hydrophilic groups, which is a property of nonpolar groups. In OPEO-type surfactant micelles, the shorter the hydrophilic chain, the stronger the interaction between the hydrophilic group and the hydroxyl group of water molecules. This is also one of the reasons that contributes to the outstanding wetting properties of the OP4 surfactant.

Finally, we compared the relative concentrations of hydrophilic and hydrophobic groups of surfactants in three modified coal systems along the *Z*-axis. The results showed that with the increase in hydrophilic chains, the relative concentration of hydrophilic head groups decreased continuously. The relative concentration of hydrophilic head groups in OP4 was the highest, reaching 25.50, while in OP9 and OP13, it was 23.77 and 21.29, respectively. In contrast, the relative concentration of hydrophobic groups of the three surfactants showed an increasing trend from 26.14 in OP4 to 28.71 in OP13. These results indicate that the adsorption capacity of the coal surface by OP4 is higher, resulting in a higher relative concentration of hydrophilic groups in the system and a denser network structure formed on the coal surface. This also makes the contact between the OP4-modified coal surface and water molecules closer and more likely to form hydrogen bonds, thereby enhancing the hydrophilicity of the anthracite.

#### 2.8.4. Mean Square Displacement

Calculating the diffusion coefficient *D* of water molecules on the surface of the modified coal allows an assessment of the effect of surfactants on the aggregation effect of water molecules on the surface of the coal [75,76]. Equations (9)–(11) are shown below:

(9) $$MSD=\frac{1}{N}\overset{N}{\underset{i=1}{\sum }}{\left[r_{i}\left(t\right)-r_{i}\left(0\right)\right]}^{2}$$

(10) $$D=\frac{1}{6N}\underset{t\to \infty }{\lim}\frac{d}{dt}\overset{N}{\underset{i=1}{\sum }}{\left[r_{i}\left(t\right)-r_{i}\left(0\right)\right]}^{2}$$

(11) $$D=\underset{t\to \infty }{\lim}\left(\frac{MSD}{6t}\right)=\frac{1}{6}K_{MSD}$$

In the above equation, MSD denotes the mean square displacement; N is the number of diffusing molecules; r(t) and r(0) denote the displacement vectors of the molecules at time *t* and *t* = 0, respectively; and $K_{MSD}$ denotes the slope curve of MSD.

The diffusion coefficients of water molecules in the OP4, OP9, and OP13 systems are 0.63835, 0.71594, and 1.25392 Å^2^/ps, respectively. Studies have shown that the coal surface adsorbed with OPEO surfactants containing more hydrophilic groups and ethylene oxide units can release more water molecules and have a higher diffusion rate. The water molecule migration rate on the OP4-modified coal surface is the lowest, indicating the strongest binding ability to water molecules and the higher hydrophilicity of the OP4-modified coal surface. This finding effectively verifies that OPEO molecules with higher ethoxylation degrees have a lower saturation adsorption density, smaller adsorption strength, less coverage on the coal surface, and worse wetting effects on anthracite coal than OPEO surfactants with short hydrophilic chains.

## 3. Experiment and Simulation

### 3.1. Experimental Materials

Anthracite is a high-rank coal with extremely poor dust wetting properties, which poses significant health and safety hazards for miners. This study focuses on anthracite obtained from the No. 3 coal seam of the Zhaozhuang coal mine in Jincheng, Shanxi Province, China. Xue’s research [77] suggests that coal dust particles exhibit a significant particle size distribution peak around 60–70 μm for inhalable dust particle size distribution. Consequently, we obtained coal powder with a size of 200 mesh (74 μm) by crushing and sieving in this study. After acid-washing with HCl/HF/HCl to reduce its ash content, the coal powder had an ash content of 0.3%. The coal powder was then vacuum-dried at 105 °C for 10 h, cooled to room temperature, and sealed for later use.

**Figure 11:**
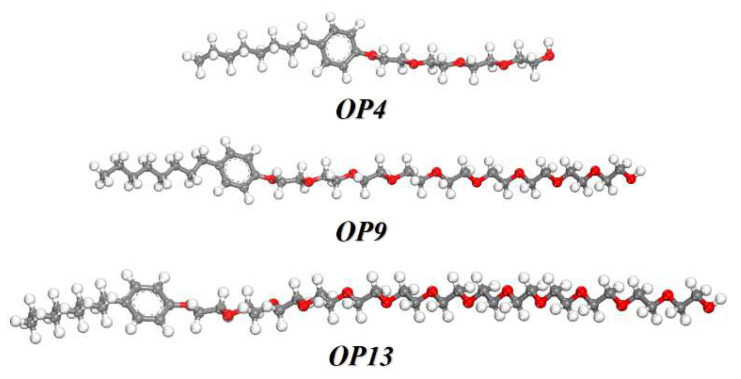
Schematic of the surfactant molecular structure.

Non-ionic surfactants are extensively researched due to their high surface activity, which significantly enhances the wetting properties of coal dust. For this study, we used the octylphenol polyoxyethylene ether series OP4, OP9, and OP13 as experimental reagents. These reagents were supplied by Jiangsu Hai’an Petrochemical Plant and had a purity exceeding 99%. Figure 11 depicts the molecular structures of these reagents. Deionized water was used as the solvent in all experiments.

### 3.2. Experimental Facilities and Parameters

#### 3.2.1. Adsorption Experiment

A mixture of 500 mg of coal sample and 500 mL of OP series solution (both at 300 mg/L) was prepared in a 600 mL beaker and held at 298.15 K for 20 h for adsorption. The resulting mixture was then separated into coal and supernatants using a centrifuge. Afterwards, the coal sample was washed with deionized water to complete the isolation process.

#### 3.2.2. Surface Tension Measurement

The surface tension of three surfactants was measured using the DCAT21 surface tension meter (Dataphysics Company, Filderstadt, Germany). Measurements were performed at 298.15 K using the drop method, which measures the gas–liquid interface.

#### 3.2.3. Contact Angle Measurement

The JY-82C contact angle measurement instrument (Chengde Testing Equipment Co., Ltd., Chengde, China) can measure the contact angle between deionized water, three surfactants, and raw coal samples without adsorption treatment. Three measurements were taken for each sample, and the average value was calculated. The volume of the surfactant droplet was controlled at 0.5 μL for each experiment. Prior to this experiment, the raw coal powder was pressed into circular specimens with a diameter of roughly 12 mm and a thickness of roughly 2 mm using an infrared press at a pressure of 20 MPa for a duration of 5 min. It is necessary to process the experimental data using the ImageJ software to improve data clarity.

#### 3.2.4. FTIR Measurement

The surface functional group content was characterized in coal samples within the range of 4000–400 cm^−1^ by means of the Thermo Scientific Nicolet iS20 Fourier Transform Infrared Spectrophotometer (Thermo Fisher Scientific, Waltham, MA, USA). The interference of water in the samples was eliminated using the KBr method prior to the experiments. Spectra correction and data processing were then carried out with the OMNIC software to enhance the accuracy and reliability of the data.

#### 3.2.5. XPS Measurement

XPS experiments were performed using the Thermo Scientific K-Alpha X-ray electron spectrometer (Thermo Fisher Scientific, USA). A monochromatic Al target (Kαhv = 1486.6 eV) was used as the X-ray source. The full-spectrum scan pass energy was 100 eV, and the resolution of the test scan was 1 eV. The narrow-spectrum scanning was performed five times with cyclic signal accumulation. The pass energy was 50 eV, and the step size was 0.05 eV, which was used for the scanning of C 1s. The binding energy was charge-corrected for the spectrum by contaminating carbon (C 1s = 284.8 eV).

#### 3.2.6. UV Spectrophotometer Measurement

The UV-3600i Plus visible-light ultraviolet spectrophotometer (Shimadzu Corporation, Kyoto, Japan) was employed to perform the ultraviolet spectroscopic analysis on centrifuged liquid and surfactants of known mass concentration. The adsorption of three surfactants on the surface of anthracite was calculated by the difference method. The spectral test was initiated at a wavelength of 200 nm and terminated at a wavelength of 800 nm, with a scan speed of 300 nm/min, a sampling interval of 0.5 nm, and a slit width of 2 nm. At 340 nm, the tungsten lamp was replaced with a deuterium lamp.

#### 3.2.7. Specific Surface Area and Porosity Distribution Test

We utilized the automated Conta Autosorb-IQ surface and pore size distribution analyzer (Conta Instruments, Boynton Beach, FL, USA) to determine the surface area and pore size distribution of both raw coal and modified coal. The Brunner–Emmet–Teller (BET) and density functional theory (DFT) methods were employed for the characterization process. N2 was used as the adsorbate in the experiment, which was carried out at 77.35 K, and the results were subsequently calculated.

#### 3.2.8. SEM Measurement

With the aid of a TESCAN MIRA LMS scanning electron microscope (TESCAN ORSAY HOLDING, a. s., Kohoutovice, Czech Republic), microstructural characterization of modified coal samples was performed to comprehend their underlying properties.

### 3.3. Molecular Dynamics Simulation

This study utilized an anthracite model previously established by our team [51,71]. The model had a crystal cell size of 5.4 × 4.8 × 5.5 nm^3^ (X·Y·Z) and a density of 1.43 g/cm^3^. The model included a surfactant layer of 10 molecules and a water layer of 2000 molecules. Molecular dynamics simulations were conducted using Materials Studio 2020 with the COMPASS force field selected for intermolecular interactions. Prior to the simulations, the Smart algorithm minimized the energy of all models. The NVT ensemble was employed for the simulations with Nose serving as the thermostat.

**Figure 12:**
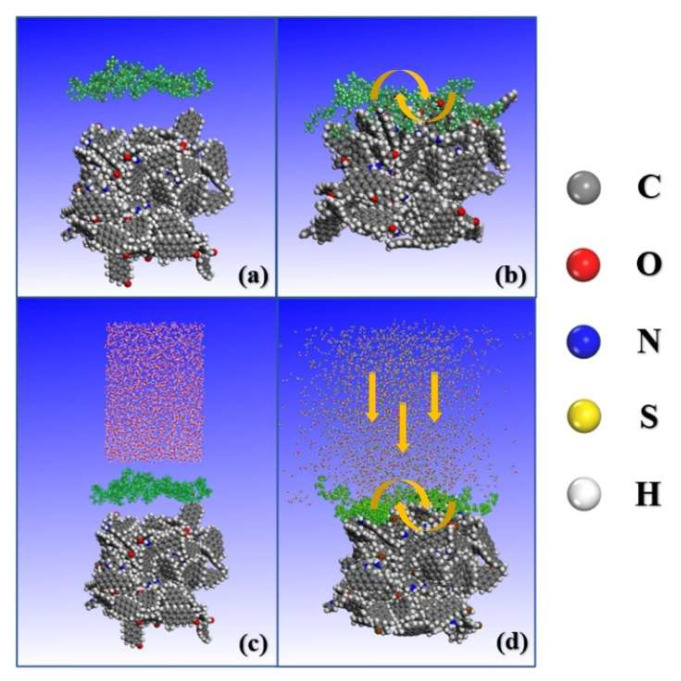
MD simulation process (OP4 as an example): (**a**) initial conformation of the first stage; (**b**) the first stage balances conformation; (**c**) initial conformation of the second stage; (**d**) the second stage balances conformation. (The colored parts are water molecules: the gray atoms are carbon atoms, red are oxygen atoms, blue are nitrogen atoms, yellow are sulfur atoms, and white are hydrogen atoms).

The first stage involved simulating a two-phase system of surfactant and anthracite undergoing a 1000 ps dynamics simulation to analyze configuration and energy changes during the adsorption process. The second stage simulated a three-phase system of water–surfactant–anthracite with similar parameters but with a 500 ps dynamics simulation to examine the water molecules’ wetting properties on the modified coal surface and the molecular escape mechanism. Long-range electrostatic interactions were calculated using the Ewald algorithm with a precision of 0.001 kcal/mol, while van der Waals interactions were calculated using the atomic algorithm with a cutoff distance of 1.25 nm. An added vacuum layer of 80 Å eliminated mirror effects on each system model surface [78]. Figure 12 depicts the absorption process during the two stages of simulations, employing OP4 as an example.

## 4. Conclusions

In this study, we evaluated the ability of OP4, OP9, and OP13 to improve the wettability of anthracite via multiple comparative experiments. We used molecular dynamics modeling to determine the micro-mechanisms of different wetting properties of OPEO surfactants.

Surface tension tests were used, and it was found that, in the absence of anthracite, OP4 had the lowest surface tension (27.182 mN/m) compared to the other two reagents. OP4 also showed significantly better adsorption performance at the water–air interface. Contact angle tests revealed that OP4 has the highest ability to improve the wettability of anthracite effectively. This was confirmed by the K value of the wetting kinetics model established. In addition, FTIR and XPS experimental results also support this by showing that OP4 is capable of introducing the most hydrophilic elements and groups to the modified coal surface.

UV spectroscopy testing revealed that OP4 had the highest adsorption amount on the coal surface, reaching 133.45 mg/g. A comparison of the specific surface area and pore distribution characteristics of coal dust before and after surfactant treatment showed that the adsorption of numerous agent molecules reduced the number and volume of pores in coal. OP4’s strong adsorption ability caused its specific surface area to increase. Moreover, the SEM analysis results demonstrated that the surfactant caused filling and aggregation phenomena, further indicating that it improves the wettability of anthracite.

Via molecular dynamics simulations, we found that the longer hydrophilic chains of OPEO surfactants do not lead to the increased adsorption of surfactant molecules on the surface of anthracite. On the contrary, excessively long hydrophilic chains tend to entangle and hinder their access to the water phase, thus reducing their ability to capture more water molecules. Conversely, shorter hydrophilic chains of the reagent can better adsorb on the coal surface via π-π interaction with the hydrophobic groups, and, since the hydrophilic chains are shorter, they are more easily extended into the water phase and not blocked by space effects. Thus, they do not restrict the adsorption of more molecules in multiple layers. Therefore, after OP4 absorbs onto the surface of anthracite coal, the polarity and water-binding capacity of the anthracite coal surface are significantly increased, resulting in a reduction in anthracite coal dust.

Our results not only contribute to a deeper understanding of the micro-mechanisms of surfactant structure in improving the wettability of anthracite coal but also provide a vital reference for the future selection and design of efficient dust suppression systems. We recommend a further discussion of the significance and application prospects of our research.

## Figures and Tables

**Table 1:** Surface tension values of reagents.

Reagents	Ethylene Oxide Number	Surface Tension (mN/m)	CMC (10^−2^ mol/L)	HLB
OP4	4	27.182	0.00223	9.4
OP9	9	33.391	0.00246	13.5
OP13	13	35.900	0.00275	14.0
Deionized water	/	71.558	/	/

**Table 2:** Fitting equations for the wetting kinetics of three surfactants.

Surfactant	Fitting Formula	K	R^2^
OP4	θ = 2020.05/(100.5 − 80.4 × exp(−0.25000 × K × t))	1.91781 ± 0.08048	0.94557
OP9	θ = 4223.68/(107.2 − 67.8 × exp(−0.58112 × K × t))	1.16375 ± 0.02970	0.97389
OP13	θ = 6880.68/(110.8 − 48.7 × exp(−1.27515 × K × t))	0.68961 ± 0.01311	0.98127

**Table 3:** C 1s and O 1s content of the anthracite surface after adsorption.

	Functional Groups	Raw Coal	OP4	OP9	OP13
C 1s	C-C/C-H (%)	88.87	68.82	74.20	75.76
	C-O (%)	4.11	23.48	21.11	16.26
	C=O (%)	7.00	0.29	2.33	1.21
	O=C-O (%)	0.02	7.41	2.36	6.77
O 1s	C-O/OH (%)	18.60	86.31	56.10	50.87
	C=O (%)	59.05	10.15	25.57	34.90
	COO/COOH (%)	22.35	3.54	18.32	14.23

**Table 4:** Calculation of the adsorption capacity of three surfactants.

Surfactant	Absorbance	*C*_1_ (mg/L)	*C*_0_ (mg/L)	*V* Liquid (L)	*m* (g)	*w* (mg/g)
OP4	1.1213	166.55	300	0.5	0.5	133.45
OP9	0.8870	227.72	300	0.5	0.5	72.28
OP13	0.4822	236.32	300	0.5	0.5	63.68

**Table 5:** Interaction energy of modified anthracite surfaces.

Model	*EV*/(kcal·mol^−1^)	*EE*/(kcal·mol^−1^)	*E*/(kcal·mol^−1^)
OP4–Anthracite	−1017.792 (95.64%)	−46.38 (4.36%)	−1064.172
OP9–Anthracite	−441.722 (86.35%)	−69.797 (13.65%)	−511.519
OP13–Anthracite	−436.561 (94.09%)	−27.392 (5.91%)	−463.953

## Data Availability

The authors do not have permission to share the data.

## Supplementary Materials

The following supporting information can be downloaded at: https://www.mdpi.com/article/10.3390/molecules28124748/s1, Figure S1. FTIR fitting curve in the range of 3700–3000 cm^−1^. Figure S2. FTIR fitting curve in the range of 3000–2800 cm^−1^.
