# Supplementary material for: Molecular Mechanism Study on the Effect of Microstructural Differences of Octylphenol Polyoxyethylene Ether (OPEO) Surfactants on the Wettability of Anthracite
Source: Molecules. 2023 Jun 13;28(12):4748. doi: 10.3390/molecules28124748 (PMC10303189; doi:10.3390/molecules28124748)
Supplement: Supplementary file 1 [file molecules-28-04748-s001.zip › molecules-2439822-supplementary.pdf]

# Supplementary Materials

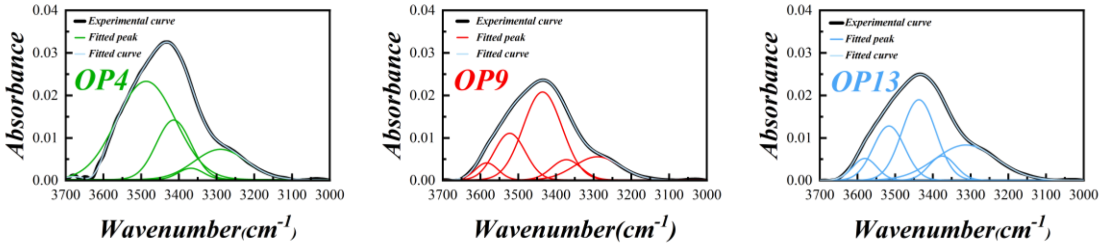


**Figure S1.** FTIR fitting curve in the range of 3700-3000 $cm^{-1}$.

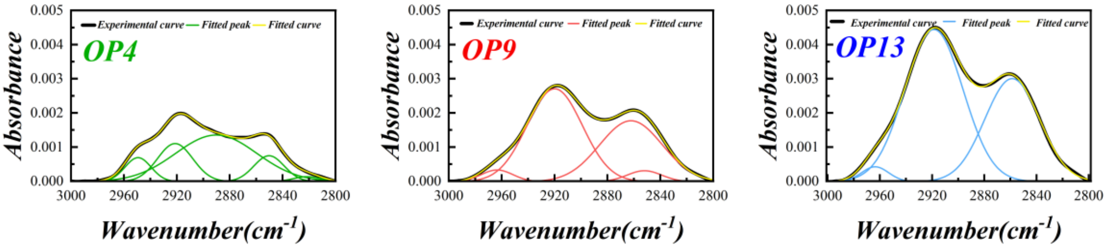


**Figure S2.** FTIR fitting curve in the range of 3000-2800 $cm^{-1}$.
